# Modifications in Macular Perfusion and Neuronal Loss After Acute Traumatic Brain Injury

**DOI:** 10.1167/iovs.64.4.35

**Published:** 2023-04-28

**Authors:** Jenny L. Hepschke, Elinor Laws, Noor H. Bin Saliman, Stefana Juncu, Ella Courtie, Antonio Belli, Richard J. Blanch

**Affiliations:** 1 Ophthalmology Department, University Hospitals Birmingham NHS Foundation Trust, Birmingham, United Kingdom; 2 Neuroscience and Ophthalmology, Institute of Inflammation and Ageing, College of Medical and Dental Sciences, University of Birmingham, Birmingham, United Kingdom; 3 NIHR Surgical Reconstruction and Microbiology Centre, University Hospitals Birmingham NHS Foundation Trust, Birmingham, United Kingdom; 4 Centre for Optometry Studies, Faculty of Health Sciences, Universiti Teknologi MARA Cawangan Selangor, Bandar Puncak Alam Selangor, Malaysia; 5 Department of Psychology, University of Portsmouth, Portsmouth, United Kingdom; 6 Neurosurgery Department, University Hospitals Birmingham NHS Foundation Trust, Birmingham, United Kingdom; 7 Academic Department of Military Surgery and Trauma, Royal Centre for Defence Medicine, Birmingham, United Kingdom

**Keywords:** traumatic brain injury (TBI), retina, optical coherence tomography (OCT), optical coherence tomography angiography (OCTA), ganglion cell layer (GCL)

## Abstract

**Purpose:**

Traumatic brain injury (TBI) causes structural damage and functional impairment in the visual system, often with retinal ganglion cell (RGC) degeneration occurring without visual symptoms. RGC degeneration is associated with reduced retinal blood-flow, however, it is not known whether reductions in perfusion precede or are secondary to neurodegeneration.

**Methods:**

We conducted a prospective observational single-center case series. Patients were included if they were admitted to the hospital after acute TBI and underwent ophthalmic clinical examination, including optical coherence tomography (OCT) and OCT angiography (OCTA) acutely and at follow-up. Ganglion cell layer thickness (GCL) thickness, vascular density in the superficial vascular plexus (SVP), and intermediate capillary plexus (ICP) were quantified.

**Results:**

Twenty-one patients aged 20 to 65 years (mean = 38 years) including 16 men and 5 women were examined less than 14 days after moderate to severe TBI, and again after 2 to 6 months. Macular structure and perfusion were normal at baseline in all patients. Visual function was abnormal at baseline in three patients and subsequent neurodegeneration and loss of perfusion corresponded to baseline visual function abnormalities. Nine patients (43%) had reduced macular GCL thickness at follow up. Perfusion in the SVP strongly associated with local GCL thickness. The strongest association of the SVP metrics was the sum of vessel density (*P* < 0.0001).

**Conclusions:**

In cases of reduced visual function after TBI, macular perfusion remained normal until reductions in GCL thickness occurred, indicating that perfusion changes were secondary to local GCL loss.

Classical traumatic optic neuropathy (TON), with clinically detectable visual loss and pupillary abnormalities occurs in 0.5% to 8% of patients after traumatic brain injury (TBI),^1^^–^^4^ although some authors have suggested this frequency to be as high as 26%.^5^

Optical coherence tomography (OCT) allows evaluation of the retina at near-histological resolution, detecting subtle neurodegenerative changes in retinal ganglion cells (RGCs) by quantifying ganglion cell layer (GCL) thickness. A related imaging modality, OCT angiography (OCTA), demonstrates flow in the retinal microstructure.^6^ When imaging patients with TON using OCT and OCTA, GCL thinning and reduced retinal perfusion, with optic atrophy evident as disc pallor, occur after 6 weeks.^1^ Recent OCT studies suggest that structural changes in the retina secondary to TBI can be subclinical, with GCL thinning and reduced retinal perfusion in up to 30% to 40% of mild TBI cases without visual symptoms^7^^,^^8^; however, the relationship between GCL thinning and reduced retinal perfusion is not well understood.

Retinal perfusion is reduced in relation to cerebral perfusion with retinal perfusion mirroring cerebral perfusion in healthy patients.^9^^–^^11^ TBI impairs cerebrovascular autoregulation and may cause progressive reductions in blood flow, leading to hypoxia.^12^ Given that vascular diseases, such as stroke and dementia, are associated with retinal microvascular changes, it is possible that the impairment of cerebrovascular autoregulation in TBI may also be associated with alterations in retinal perfusion.^6^^,^^13^ As both retinal and brain perfusion are locally regulated in response to resting metabolic demand,^9^^–^^11^ it is also possible that reductions in retinal perfusion occur in response to reductions in RGC function, immediately after injury, rather than 4 to 6 weeks later when neurodegeneration occurs.

Retinal perfusion is reduced in local vascular disease, such as anterior ischemic optic neuropathy, where optic nerve head hypoperfusion precedes and causes RGC degeneration.^14^ The RGC degeneration and reduced retinal perfusion also occur in chronic pathologies without local vascular compromise, such as Alzheimer's disease, multiple sclerosis (MS), glaucoma, and compressive optic neuropathy, which feature both RGC degeneration and reduced retinal perfusion on OCTA.^14^^–^^16^ It is not clear whether, in patients with acute RGC damage, reductions in retinal perfusion precede the RGC degeneration (potentially functioning as an early marker of injury), or occur secondary to it.

We therefore assessed GCL thickness and retinal vascular changes using OCT and OCTA in subjects after acute TBI to assess whether vascular changes preceded or followed neuronal degeneration, hypothesizing that reduction in perfusion would precede and predict neurodegeneration.

## Methods

### Institutional Approval

Prospective data were collected on patients with TBI as part of a service evaluation registered with the Clinical Governance Department, and data analyses for research were approved by the Health Research Authority and by the Research and Development Department at University Hospitals Birmingham NHS Foundation Trust. Control patients were recruited under: Defining Outcome Measures in Ocular Inflammatory Disease: 14/EM/1163; Ophthalmic and Neurocognitive Assessment in the Management of Critically Ill Patients: 19/YH/0113; and Eye Assessments in Traumatic Brain Injury: 20/WM/0178.

### Standards of Inclusion and Exclusion

Inclusion criteria for the wider service evaluation dataset were adult patients diagnosed with acute TBI between October 2020 and October 2021 and able to cooperate with OCT examination. Patients were included in the retrospective analysis if they had OCTA <14 days after injury (acute) and at follow-up between 6 weeks and 6 months after injury. The acute time point was chosen to occur before RGC loss is detectable on OCT, and the follow-up time point after optic atrophy occurs in TON.^1^

Patients were excluded from the data analysis if they had any direct penetrating or nonpenetrating injury to the eye, any pre-existing optic nerve or retinal pathology, or if they were unable to cooperate with the examination. Only patients with complete datasets for both eyes were included.

Control patients were included if they had no history of eye or brain disease and were within 5 years of age of an unmatched patient with TBI included in the study.

### Data Collection

Data collected in addition to OCT and OCTA scans included age, sex, cause of trauma, neuroradiological findings, severity of TBI, ophthalmic examination findings, presence of visual symptoms at acute visit, and time to acute and follow-up assessment.

TBI severity was based on the US Department of Defense (DoD) classification.^17^ The DoD classifies TBI into mild, moderate, and severe categories according to duration of loss of consciousness, duration of alteration in mental state, duration of post-traumatic amnesia, Glasgow coma scale assessment at time of injury, and any structural abnormalities on brain imaging (either computed tomography [CT] or magnetic resonance imaging [MRI]). ^1^Table 1^3^ is a summary of the DoD classification of TBI.^17^

**Table 1.** Summary of DoD TBI Classification^17^

Criteria	Mild	Moderate	Severe
Structural imaging	Normal	Normal or abnormal	Normal or abnormal
Loss of consciousness (LOC)	0–30 minutes	>30 minutes <24 hours	>24 hours
Alteration of consciousness/mental state	A moment up to 24 hours	>24 hours	>24 hours
Post-traumatic amnesia	0–1 day	>1 and <7 days	>7 days
Glasgow coma scale (best available score in first 24 hours after injury)	13–15	9–12	<9

**Table 2.** Baseline Characteristics of Included and Excluded Participants and Controls

Characteristic	Included Participants			Control Group		*P* Value (SD) Included Baseline Versus Controls	
Age	Mean = 38 years (range = 19-64 years)			Mean = 44 years (range = 17-65)		0.102 (3.16)	
Sex	16 M, 5 F			11 M, 7 F		0.309	
Visual acuity LogMAR (Snellen)	Median: 0 (20/20)			N/A		N/A	
Mechanism	Fall: 8/21 (28.6%)			N/A		N/A	
	Assault: 6/21 (28.6%)				
	RTC: 7/21 (42.9%)				
	Sport: 0/21 (0%)				
Injury severity	Moderate 16/21 (76%)			N/A		N/A	
	Severe 5/21 (24%)				
Time after injury of first visit (days)	Mean = 5 days (SD = 2.9 days)			N/A		N/A	
Time after injury of second visit (days)	Mean = 146.7 days (SD = 45.1 days)			N/A		N/A	
Mean (SD) GCL thickness in each EDTRS grid sector	<14 days		>/=14 days				
	C0	13.95 (3.35)	13.4 (3.45)	C0	14.45 (2.72)	C0	0.238 (0.76)
	I1	50.48 (5.77)	49.25 (7.27)	I1	51.5 (3.91)	I1	0.147 (1.24)
	T1	47.08 (5.98)	45.53 (7.53)	T1	47.05 (5.24)	T1	0.382 (1.39)
	S1	51.00 (5.87)	50.00 (6.69)	S1	51.54 (4.39)	S1	0.584 (1.30)
	N1	49.85 (5.46)	47.95 (7.65)	N1	51.22 (3.60)	N1	0.056 (1.16)
	I2	33.68 (3.68)	32.93 (3.81)	I2	32.86 (2.92)	I2	0.151 (0.83)
	T2	35.53 (3.87)	34.93 (4.38)	T2	36.27 (3.86)	T2	0.832 (0.95)
	S2	34.55 (3.00)	33.85 (4.06)	S2	35.41 (3.70)	S2	0.207 (0.81)
	N2	38.1 (3.75)	13.4 (3.45)	N2	38.36 (3.90)	N2	0.861 (0.94)
Mean (SD) perfusion (SVP perfusion density) in each EDTRS grid sector	<14 days		>/=14 days				
	C0	7.71 (4.18)	7.17 (3.87)	C0	6.32 (3.65)	C0	0.487 (0.96)
	I1	32.03 (7.82)	32.85 (7.17)	I1	30.89 (8.90)	I1	0.725 (2.03)
	T1	29.02 (7.29)	31.44 (5.78)	T1	28.64 (8.21)	T1	0.646 (1.89)
	S1	31.83 (7.47)	33.05 (6.89)	S1	30.82 (9.76)	S1	0.089 (2.09)
	N1	31.90 (7.79)	34.75 (6.80)	N1	30.81 (8.79)	N1	0.570 (2.02)
	I2	27.67 (6.27)	27.14 (4.96)	I2	27.21 (5.42)	I2	0.287 (2.47)
	T2	26.60 (5.73)	27.55 (4.98)	T2	25.82 (7.61)	T2	0.237 (2.48)
	S2	30.07 (5.59)	29.47 (6.23)	S2	27.95 (6.42)	S2	0.344 (2.34)
	N2	33.52 (6.23)	33.16 (5.59)	N2	31.82 (3.39)	N2	0.283 (2.34)

TBI, traumatic brain injury; RTC, road traffic collision; GCL, ganglion cell layer; ETDRS, macular area; SVP, superficial vascular plexus.

**Table 3.** Effect of TBI on GCL Thickness at Follow-Up

	Included Participants (With OCTA)	
	Number (Proportion)	Mean (SD) GCL Thickness Change (*Across Affected Sectors)
Unilateral severe traumatic optic neuropathy	1/21 (5%)	−26.6 (13.34)*
Homonymous GCL loss	2/21 (10%)	−7.9 (4.03)*
GCL loss in at least one sector	6/21 (29%)	−5.3 (2.15)*
No GCL loss above 3 µm	12/21 (57%)	+0.3 (2.05)

Loss of GCL thickness >3 µm between the acute OCT measurement and follow up was classified as a detectable change.

### OCT/OCTA Image Acquisition

OCT and OCTA images were acquired using the SPECTRALIS Heidelberg OCT2 table-top module (Heidelberg Engineering, Heidelberg, Germany). Each OCT volume consisted of the manufacturer's “Posterior Pole” protocol to acquire volumetric retinal scans in 61 horizontal B-scans averaged over at least 9 frames in a 30 degrees × 25 degrees fovea-centered volume. Each OCTA volume consisted of 521 B-scans with an interscan distance of 1.79 µm nominal spacing in a 6 × 6 mm total area. Seven repeated OCT B-scans at each tissue location were used by the full-spectrum probabilistic OCTA algorithm to determine the presence or absence of flow at each voxel.

Measurements of GCL thickness in the macula within the Early Treatment of Diabetic Retinopathy Study (ETDRS) grid areas were obtained using manufacturer's software (Heidelberg Eye Explorer, version 1). RGC loss after TBI between baseline and follow-up was classified on OCT as: “no change” where GCL loss was ≤3 µm,^18^ “decline” in cases of GCL loss >3 µm in any ETDRS sector, and “severe decline” with GCL loss >8 µm in any ETDRS sector.

OCTA data were exported as .E2E files for further analysis using manufacturer's software (SP-X1902; Heidelberg Engineering, Heidelberg, Germany). The onboard volumetric PAR algorithm (“3D PAR vessel strong”) that performs 3D vessel shape estimation in the volume and only operates to remove their projection tails was used to remove projection artifacts. Serial follow-up OCT and OCTA scans were co-registered on the basis of the fundus image ensuring that exactly the same area is imaged on repeat scans. As perfusion is measured at a single point in time and averaged across the temporal window of the scan time, a variability measure is not provided.

The macula was divided into the ETDRS grid areas and perfusion analysis within individual sectors compared with GCL thickness. The ETDRS grid areas are C0 (foveal), S1 (inner and superior), S2 (outer and superior), T1 (inner and temporal), T2 (outer and temporal), I1 (inner and inferior), I2 (outer and inferior), N1 (inner and nasal), and N2 (outer and nasal). Software-calculated perfusion metrics were exported in the superficial vascular plexus, intermediate capillary plexus, and deep capillary plexus (SVP, ICP, and DCP, respectively): Mean, sum, probability (prob), skeletonized vascular density (skeleton), perfusion density, vessel length density, vessel enhanced perfusion density (vessel enhanced), and large vessel masked flow sum (LVMS) and probability (LVMP).

These metrics evaluate: mean – expected probability of OCTA signal for a pixel in the vascular layer; sum – amount of OCTA signal within the vascular layer; prob – probability that OCTA signal exists in the vascular layer; skeleton – density of vessel center lines; vessel enhanced – scaled version of probability output to suppress noise while preserving small vessels; LVMS/LVMP – sum/probability output, respectively, with large vessels removed from the analysis; perfusion density – measured in a binarized image; and vessel length density – derived from skeletonized vascular density using manufacturer's formula.^19^ These perfusion metrics may be divided as: skeletonized (skeleton and vessel length density), which measure vessel length only; binarized (perfusion density), which collapse all blood flow data to either 0 or 1; and scaled methods (mean, sum, prob, vessel enhanced, LVMS, and LVMP), sensitive to different levels of blood flow within the vessels on a continuous 32-bit scale between 0 and 1.

### Statistical Analysis

The results were analyzed using R software. Perfusion was modeled as a dependent variable using linear regression, GCL thickness as a covariate, and time point after injury, and eye and retinal area (in ETDRS grid) as predictor variables. A value of *P* < 0.05 was considered statistically significant. Measures from right and left eyes were modeled as repeated measures on the same subject at each time point (where time point was also a within-subject repeated measures factor). Partial squared eta (η^2^p) was used to assess the proportion of variability in the dependent variable explained by individual independent variables in the model. In general, eta squared is calculated the same way as R squared (i.e. out of the total variation in Y, the proportion that can be attributed to a specific X) and has values between 0 and 1 (similar to R squared). As more variables are added, each individual eta squared will reduce as each variable will explain the some of the variance. We have used partial eta squared where any variation explained by other variables is removed from the denominator. It is generally considered that η^2^ = 0.01 indicates a small effect; η^2^ = 0.06 indicates a medium effect; and η^2^ = 0.14 indicates a large effect. Control, excluded, and included patients were compared using the *t*-test for continuous data and chi-squared test for proportions.

### Data Availability

Raw data were generated at and are owned by the University Hospitals Birmingham NHS Foundation Trust. Derived data supporting the findings of this study are available from the corresponding author on reasonable request, subject to a data transfer agreement.

## Results

### Baseline Participant Characteristics

Patient characteristics at entry into the study are shown in Table 2 in comparison to the control group and in comparison to the wider TBI cohort in the Supplementary Material. Twenty-one patients with acute TBI were included from the wider TBI cohort who had an acute OCT/OCTA performed <14 days after injury (mean = 5 days, SD = 2.94) and follow-up assessment between 2 and 6 months after injury (mean = 145 days, SD = 45.23). The wider TBI cohort were on average younger (*P* = <0.001), had a higher percentage of men, overall lower severity of TBI (*P* = <0.001), and higher proportion resulting from sports injury (*P* = <0.001) when compared to the patients who were included in the study.

In our cohort, isolated hemorrhagic contusions were present in 4 of 21 (19%) subjects, skull/orbital or cervical vertebral fractures in 8 of 21 (39%), and intracranial hemorrhages, such as subdural (SDH), subarachnoid (SAH), and intraparenchymal/cerebral hemorrhages (ICH) were present in 9 of 21 (43%; see Table 2). Overall, 76% (16/21) patients were classified as moderate and 24% (5/21) severe TBI based on the DoD classification (see Table 2).^17^

A normal clinical examination, with visual acuity of 20/20 or better, normal color vision, pupils, and confrontational fields, was present in 86% patients (18/21) at the acute assessment stage (see Table 2).

### OCT and OCTA Changes After TBI

Comparison among the 21 included participants and the wider screened cohort within the service evaluation and the control group did not reveal a significant difference in GCL thickness or retinal perfusion (Supplementary Material) acutely after injury.

One patient had a unilateral severe TON, which was clinically apparent in the acute visit as a relative afferent pupillary defect (RAPD) and unilateral no light perception (NLP). Two patients had homonymous field loss to confrontation on acute assessment. In all 3 of 21 (14%) patients with abnormal visual assessment in the acute visit, there was severe GCL thinning at follow-up with a mean GCL thickness change of −20 µm for the patient with TON, and –8 µm for the patients with homonymous loss (Table 3). A further 6 of 21 (29%) patients showed a significant decline in GCL thickness in one or more ETDRS sector (mean reduction in GCL thickness in those 6 patients with at least one sector classed as significant decline was −6.3 µm). In our cohort, the mean time for follow-up in those with GCL loss was 145 days. The mean time to follow-up for those without GCL loss was 148 days. In the total, of nine patients classified as having significant decline in GCL thickness, seven had cortical injuries (1 extradural hemorrhage [EDH], 4 subarachnoid hemorrhage [SAH], 1 subdural hematoma [SDH], and 1 contusion). The remaining two of nine patients with significant decline in GCL thickness had skull fractures but no cortical injury evident on brain imaging. Of those without significant decline in GCL thickness, 10 of 11 patients had cortical injuries (1 EDH, 1 SDH, 1 intracranial hemorrhage, 4 had contusions, and 3 had multiple cortical injuries). Overall, 9 of 21 (43%) patients of our cohort showed a reduction in GCL thickness in the first 6 months after acute TBI (Table 3). The remaining 12 of 21 (57%) patients had no significant GCL decline with 4 of 21 (10%) patients showing GCL thickening.

**Figure 1.**
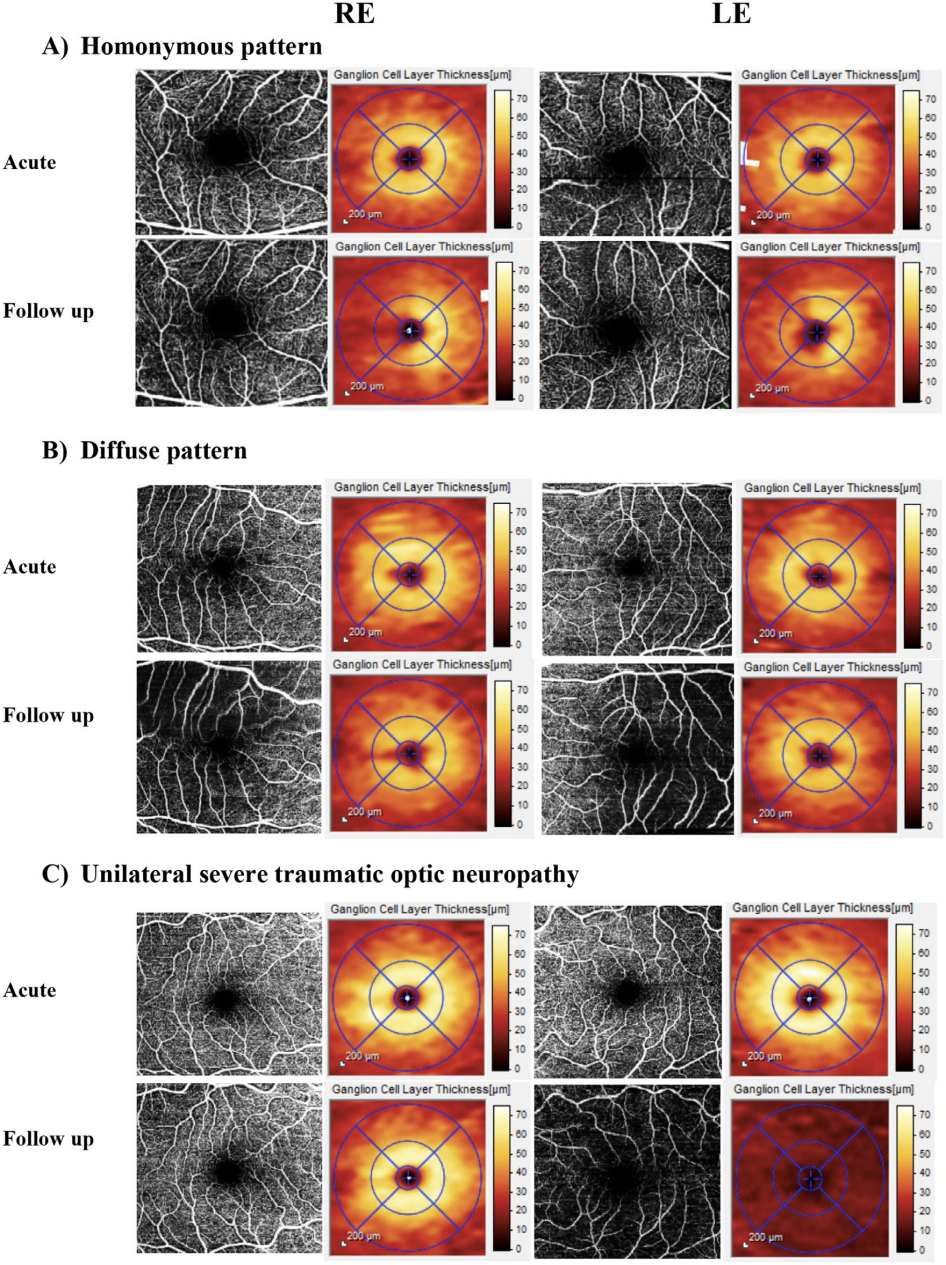
Representative examples of the patterns of neurodegeneration in the 15 degrees central macular map of the superficial vascular plexus (SVP) and en face macular ganglion cell map with EDTRS grid. (**A**) A right homonymous loss: normal GCL thickness and perfusion acutely (*top panel*) and right homonymous reduction in GCL thickness in both eyes with matching reduction in perfusion at follow-up (*bottom panel*); (**B**) global decline: normal GCL thickness and perfusion acutely (*top panel*) and reduction in the GCL thickness throughout the EDTRS sectors in both eyes more pronounced in the outer EDTRS segments and reduced microvascular perfusion at follow up (*bottom panel*); (**C**) severe traumatic optic neuropathy: normal GCL and perfusion scans acutely (*top panel*) and marked reduction of GCL thickness and vascular perfusion in the left eye at follow up (*bottom panel*). Acute time point <10 days; follow-up 2 to 6 months.

**Figure 2.**
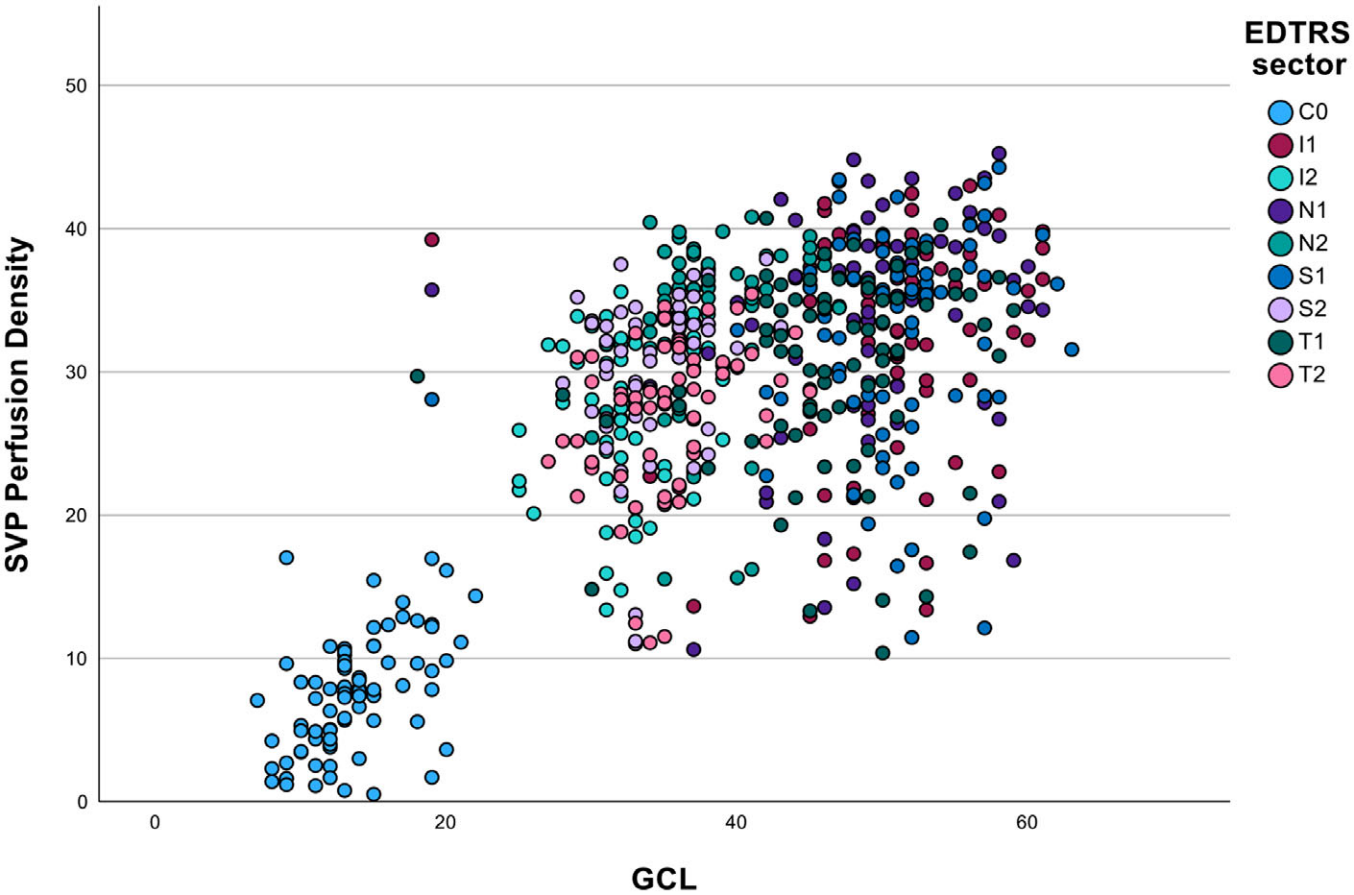
Scatter plot of superficial vascular plexus (SVP) perfusion density against ganglion cell layer (GCL) thickness divided by early treatment of diabetic retinopathy (EDTRS) grid sector showing correlation with *P* < 0.0001 and η^2^p = 0.051 for GCL and η^2^p = 0.282 for EDTRS grid sector. C0 central area; I1 inner inferior, I2 outer inferior, N1 inner nasal, N2 outer nasal, S1 inner superior, S2 outer superior, T1 inner temporal, and T2 outer temporal.

**Figure 3.**
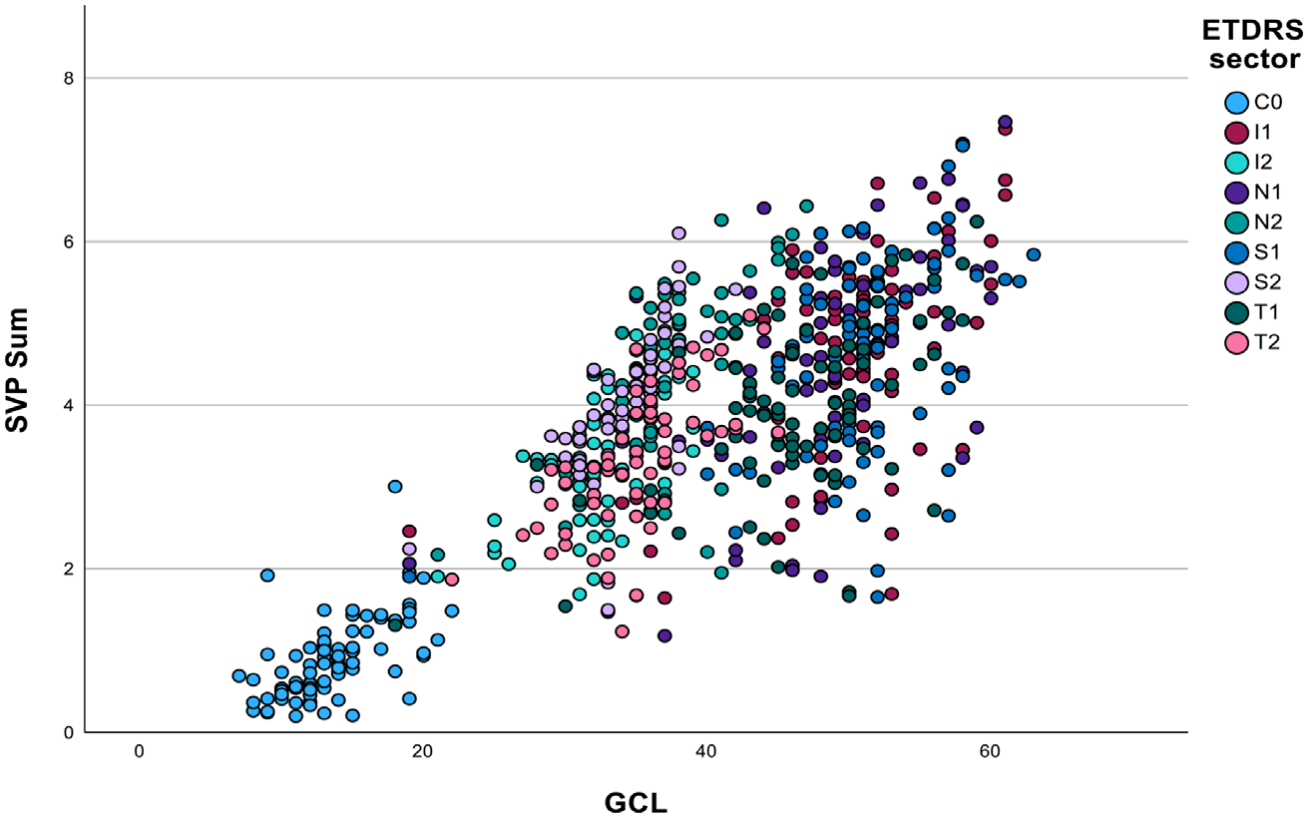
Scatter plot of superficial vascular plexus (SVP) sum against ganglion cell layer (GCL) thickness divided by early treatment of diabetic retinopathy (EDTRS) grid sector showing strong correlation with *P* < 0.0001 and η^2^p = 0.323 for GCL and η^2^p = 0.205 for EDTRS grid sector. C0 central area; I1 inner inferior, I2 outer inferior, N1 inner nasal, N2 outer nasal, S1 inner superior, S2 outer superior, T1 inner temporal, and T2 outer temporal.

Reductions in retinal perfusion in the SVP mirrored GCL thickness loss, corresponding to baseline abnormalities of visual function where these existed (Fig. 1). SVP measures were all strongly correlated with local GCL thickness, with the strongest associations being SVP sum, mean, prob, vessel enhanced, LVMS, and LVMP (Table 4, Figs. 2 and 3).

**Table 4.** Correlation of OCTA Perfusion Metrics With Macula Ganglion Cell Layer Thickness in the Superficial Vascular Plexus (SVP) and Intermediate Capillary Plexus (ICP)

	SVP		ICP		DCP	
OCTA Metric	*P*	η^2^p	*P*	η^2^p	*P*	η^2^p
Sum	<0.001	0.323	0.032	0.007	0.004	0.013
Mean	0.001	0.017	0.012	0.011	0.042	0.007
Probability	<0.001	0.292	0.018	0.009	0.008	0.012
Skeleton	<0.001	0.022	0.145	0.003	0.926	<0.001
Vessel enhanced	<0.001	0.048	<0.001	0.020	0.082	0.005
Large vessel masked sum	<0.001	0.275	0.005	0.013	0.001	0.014
Large vessel masked probability	<0.001	0.218	0.011	0.011	0.001	0.017
Perfusion density	<0.001	0.051	<0.001	0.028	0.074	0.007
Vessel length density	<0.001	0.032	<0.001	0.016	0.035	0.007

*η^2^p*, partial squared eta.

ICP perfusion was significantly correlated with GCL for many measures, but η**^2^**p was close to zero across all measures demonstrating a weaker relationship (see Table 4).

## Discussion

We present the largest study to date of patients with moderate to severe TBI with acute and follow-up ophthalmic assessments. We have shown for the first time that macular SVP retinal perfusion after anterior visual pathway damage reduces in proportion to the structural loss of macular RGC, rather than directly in response to injury, which may occur independently of detectable changes in visual function.

TBI causes structural and functional damage to the central nervous system (CNS) with primary neural and vascular structural injury.^4^^,^^20^ Traumatic cerebral vascular injury is thought to be a universal phenotype in TBI and studies have sought to explore its relationship with clinical outcomes.^21^ Initial hypoperfusion in the first 24 hours after TBI is followed by hyperemia from 1 to 3 days after injury and then vasospasm causing tissue hypoxia.^12^ Reduced cerebral perfusion was linked with poor outcomes after TBI,^22^ and secondary injury after TBI is exacerbated by impaired cerebrovascular auto-regulation and vascular permeability with triphasic blood-brain barrier breakdown.^23^

The eye and brain share embryological tissue origins. Retinal microvascular changes reflect cerebrovascular changes in neurodegenerative diseases, such as stroke and dementia, suggesting that retinal perfusion may mirror cerebral perfusion after TBI.^6^^,^^24^^,^^25^ However, in our patients, and in comparison to age-matched control patients, there was no difference in baseline retinal perfusion in any vascular plexus, which may reflect the pooling of data within the first 14 days, thus spanning all 3 phases of vascular dysregulation after TBI.

GCL thickness is used as a marker for optic nerve health with GCL thinning the final common and irreversible result of optic nerve injury.^26^^–^^28^ RGC density is highest in the macula and is relatively unaffected by acute optic nerve head changes, such as edema, in comparison to the retinal nerve fiber layer (RNFL) thickness, making it the optimal location to detect early damage.^29^ Thinning of the GCL after TBI is often mild and patchy.^26^ Observational reports and animal models demonstrate that GCL thinning is detectable 2 to 4 weeks after primary optic nerve injury,^30^^,^^31^ and after 2 months secondary to trans-synaptic degeneration.^1^^,^^27^^,^^28^

Although TON is reported in 0.5% to 8% of patients with TBI, OCT and field abnormalities may be detectable in up to 53% of patients with TBI even without detectable deficits in visual acuity.^2^^–^^4^^,^^20^ Forty-three percent of our patients had moderate to severe GCL thinning on OCT at follow-up. One patient had a severe indirect TON that showed normal GCL and OCTA metrics acutely, with structural loss only apparent at the follow-up time point, as previously predicted.^32^ In contrast, in chronic compressive optic neuropathies secondary to chiasmal compression and in glaucoma, GCL thinning may be detected within the retina before a field defect is present.^33^^,^^34^ RNFL thinning, GCL thinning, and permanent reduction in vascular caliber size and density relate topographically to optic tract changes and visual field loss in these more chronic conditions^35^^–^^37^; however, because these studies looked at chronic disease processes, they could not separate GCL loss and perfusion changes.

RGCs are situated in the inner retina, closely associated with the SVP, whereas the ICP and DCP bracket the inner nuclear layer.^8^ The cells of the inner nuclear layer do not degenerate in TON and therefore ICP and DCP perfusion would not be expected to reduce or to relate to GCL thickness after TBI, consistent with the observed weak association between GCL thickness and ICP metrics. We also used a variety of OCTA analysis methods to quantify perfusion in each vascular layer. In our study, all SVP metrics except skeletonized vascular density associated strongly with GCL thickness, with SVP sum the strongest association.

The weaker association between GCL and the skeletonized perfusion metrics could be explained by the fact that skeletonization estimates a single vessel line in the center of a vessel, reducing each vessel to a single pixel width. Changes in retinal perfusion causing changes in vessel caliber will therefore not be detected on skeletonized analysis. The SVP has larger vessels as well as capillaries. For large vessels, the software occasionally maps two parallel lines on either side of the vessel, instead of one line in the center of the vessel. Software can also have difficulty skeletonizing capillary vessels in the presence of larger vessels, which is significant for the SVP where there are both large and small vessels. Skeletonized vessel length density may therefore be less reliable in the SVP than the ICP. The skeleton metric is also sensitive to scan quality, working poorly in the presence of artifacts.

This study has several limitations. It was conducted as a single center retrospective review of patients included in a prospective clinical study. A larger prospective study could confirm the results, although the strength of the observed associations suggests that these are robust. Our inclusion criteria required patients to be able to cooperate with OCT imaging within 14 days of injury, and although 5 patients in our cohort had severe TBI, it would have excluded patients with more severe injury and marked vascular dysregulation who remained in intensive care. We were unable to relate the retinal findings to cerebral perfusion, which would be of interest particularly in severely injured patients. We also only examined two relatively broad time points designed to capture images before and after GCL loss had occurred and it would be of interest to conduct more frequent assessments in the first 12 weeks after injury.

We show that retinal GCL thickness and SVP density are normal after acute TBI, even in the context of acute reductions in visual function. Reduction in retinal perfusion after TBI follows rather than precedes GCL loss.

## Supplementary Material

Supplement 1
