# Supplementary material for: Modifications in Macular Perfusion and Neuronal Loss After Acute Traumatic Brain Injury
Source: Invest Ophthalmol Vis Sci. 2023 Apr 28;64(4):35. doi: 10.1167/iovs.64.4.35 (PMC10150830; doi:10.1167/iovs.64.4.35)
Supplement: Supplement 1 [file iovs-64-4-35_s001.pdf]

Supplementary Material

Characteristic	Excluded participants with TBI	P value (s.d.) included baseline vs excluded participants with TBI
Age	Mean 29 years (range 18-49)	<0.001 (3.62)
Sex	24 Male, 1 Female	0.047
Visual acuity LogMAR (Snellen)	Median: -0.04 (20/18)	0.199 (0.07)
Mechanism	Fall: 2/25 (8%)<br>Assault: 5/25 (20%)<br>RTC: 3/25 (12%)<br>Sport: 15/25 (60%)	<0.001
Injury severity	Mild 16/25 (64%)<br>Moderate 9/25 (36%)	<0.001 77%<br>16%
Time after injury of first visit (days)	Mean 7 days (s.d. 3.3)	0.03 (0.68)
Time after injury of second visit (days)	n/a	n/a
Mean (s.d.) GCL thickness in each EDTRS grid sector	<14 days<br>C0 14.74 (4.615)<br>I1 55.16 (4.44)<br>T1 51.92 (4.69)<br>S1 55.52 (4.62)<br>N1 53.72 (4.69)<br>I2 35.24 (3.27)<br>T2 37.80 (3.84)<br>S2 36.22 (3.28)<br>N2 40.32 (3.58)	C0 0.01 (0.86)<br>I1 0.229 (1.08)<br>T1 0.25 (1.13)<br>S1 0.76 (1.11)<br>N1 0.473 (1.08)<br>I2 0.474 (0.71)<br>T2 0.847 (0.82)<br>S2 0.645 (0.67)<br>N2 0.66 (0.78)
Mean perfusion (SVP Sum) in each EDTRS grid sector	n/a	n/a
